# Evaluation of frozen tissue-derived prognostic gene expression signatures in FFPE colorectal cancer samples

**DOI:** 10.1038/srep33273

**Published:** 2016-09-14

**Authors:** Jing Zhu, Natasha G. Deane, Keeli B. Lewis, Chandrasekhar Padmanabhan, M. Kay Washington, Kristen K. Ciombor, Cynthia Timmers, Richard M. Goldberg, R. Daniel Beauchamp, Xi Chen

**Affiliations:** 1 Vanderbilt University, Department of Surgery, Nashville, 37232, USA; 2 Vanderbilt University, Vanderbilt Ingram Cancer Center, Nashville, 37232, USA; 3 Vanderbilt University, Department of Pathology, Nashville, 37232, USA; 4 The Ohio State University Comprehensive Cancer Center, Columbus, 43210, USA; 5 Ohio State University, Division of Medical Oncology, Department of Internal Medicine, Columbus, 43210, USA; 6 Vanderbilt University, Department of Cell and Developmental Biology, Nashville, 37232, USA; 7 Vanderbilt University, Department of Cancer Biology, Nashville, 37232, USA; 8 University of Miami Miller School of Medicine, Division of Biostatistics, Department of Public Health Sciences, Miami, 33136, USA; 9 University of Miami Miller School of Medicine, Sylvester Comprehensive Cancer Center, Miami, 33136, USA

## Abstract

Defining molecular features that can predict the recurrence of colorectal cancer (CRC) for stage II-III patients remains challenging in cancer research. Most available clinical samples are Formalin-Fixed, Paraffin-Embedded (FFPE). NanoString nCounter^®^ and Affymetrix GeneChip^®^ Human Transcriptome Array 2.0 (HTA) are the two platforms marketed for high-throughput gene expression profiling for FFPE samples. In this study, to evaluate the gene expression of frozen tissue-derived prognostic signatures in FFPE CRC samples, we evaluated the expression of 516 genes from published frozen tissue-derived prognostic signatures in 42 FFPE CRC samples measured by both platforms. Based on HTA platform-derived data, we identified both gene (99 individual genes, FDR < 0.05) and gene set (four of the six reported multi-gene signatures with sufficient information for evaluation, P < 0.05) expression differences associated with survival outcomes. Using nCounter platform-derived data, one of the six multi-gene signatures (P < 0.05) but no individual gene was associated with survival outcomes. Our study indicated that sufficiently high quality RNA could be obtained from FFPE tumor tissues to detect frozen tissue-derived prognostic gene expression signatures for CRC patients.

CRC is the second leading cause of cancer-related deaths in the United States when both sexes are combined^1^. For patients diagnosed with localized disease, the 5-year relative survival rate is relatively high at 90.3%. However, after the cancer has spread regionally to involve adjacent organs or lymph nodes (stage III), the 5-year survival rate drops to 70.4%^2^. Randomized clinical trials have shown survival benefit for stage III colon cancer patients treated with adjuvant chemotherapy^3^ but not consistently for stage II colon cancer patients^4^. However, in these trials, a substantial subset stage III colon cancer patients if treated by surgery alone would not recur in five years^5^ and it also appears that a subset of high-risk stage II colon cancer patients may benefit from adjuvant treatment^6^.

To identify high-risk stage II CRC patients and low-risk stage III CRC patients, researchers have studied a substantial number of molecular biomarkers over the past decade. For single gene or tumor phenotype biomarker, microsatellite instability (MSI) high is a validated prognostic biomarker in early stage colorectal cancer while the prognostic value of *BRAF* or *KRAS* mutation depends on microsatellite status and tumor location^7^. However, only approximately 15% of early-stage CRC patients are MSI high^8^.

In addition to single gene biomarkers, the prognostic value of multi-gene signatures from supervised gene expression analysis has been widely evaluated. OncotypeDX Colon Cancer^9^, ColoPrint^10^, Veridex^11^ and GeneFx Colon^12^ are signatures that have been evaluated in independent studies. However, a recent study by Di Narzo, A. *et al.*^13^ showed that these gene expression-based risk scores provide prognostic information but add little additional clinical value to the established risk factors of T-stage, N-stage and MSI status. Moreover, contradictory results relating to low or high risk were observed for individual patients when applying the four different risk scores^13^.

To identify novel prognostic signatures in future studies, gene expression profiling using FFPE samples is needed to facilitate studies of much larger sample sizes, since most available clinical samples are FFPE preserved samples. Moreover, a number of prognostic gene expression signatures have been derived from fresh frozen tissues^10^,^14^,^15^,^16^,^17^,^18^,^19^,^20^,^21^,^22^,^23^,^24^,^25^,^26^,^27^,^28^,^29^,^30^,^31^. To make use of this valuable data resource for assisting with novel prognostic signature development in the future studies, it will be necessary to re-evaluate these gene expression signatures in FFPE samples.

Nanostring nCounter^®^^32^ and Affymetrix GeneChip^®^ Human Transcriptome Array 2.0 (HTA)^33^ are two available platforms that enable gene expression profiling of FFPE samples. The nCounter platform focuses on customized targeted gene expression profiling while the HTA platform measures the genome-wide gene expression. In this study, 516 genes derived from multiple published frozen tissue-derived CRC prognostic signatures were evaluated for their prognostic value based on the gene expression patterns in RNA extracted from FFPE CRC primary tumor samples and measured by both platforms.

## Results

### Sample collection

In total, a dataset of 500 stage II or III CRC patients with archived tumor samples and complete pathological information was obtained from the Vanderbilt Translational Pathology and Imaging Core. Patients with stages I and IV were excluded from the dataset and other patient samples were excluded if one of the following criteria was present: (1) if metastatic disease was documented within 3 months of resection of the primary CRC; (2) non-adenocarcinoma tumor histology; (3) history of chemotherapy administered prior to surgical resection; (4) insufficient availability of electronic medical records (lack of adequate documentation of operative report, pathology report and correct medical record number); (5) unavailable or inadequate tumor specimens (including lack of tumor in sample or <80% tumor cells on the tissue sections cut from the FFPE block and samples not of the primary resection); (6) inadequate clinical follow-up (at least 3 years postoperative follow-up if no recurrence); (7) failure of RNA extraction and (8) highly degraded (less than 20% of the RNA sample containing fragments of at least 300 base pairs in length). Ultimately, we identified 194 acceptable primary stage II and stage III FFPE preserved CRC specimens and these specimens were associated with a 7.4-year mean patient follow up.

### nCounter platform development

A custom nCounter^®^ assay (Nanostring Technologies, Seattle, WA) was designed for quantitative assessment of expression of 536 gene elements. We included gene elements from 27 published and 6 unpublished signature gene lists related to CRC recurrence, CRC metastasis and colonic stem cells^10^,^14^,^15^,^16^,^17^,^18^,^19^,^20^,^21^,^22^,^23^,^24^,^25^,^26^,^27^,^28^,^29^,^30^,^31^, selecting those that were presented in ≥2 gene lists. In total, 460 genes were present in at least 2 gene lists and 459 out of these 460 were from published gene lists. All these genes along with their tissue origin, platform initially used for signature identification and the relevance to the current study were listed in Supplementary Table S1. Additional 69 candidate genes of interest related to epithelial-mesenchymal transition and the study of metastatic behavior in cancer cells (e.g., CDH1) were also added to the assay. The final codeset contains 536 genes (Supplementary Table S2) including seven housekeeping genes with the least variant expression elements in our pilot nCounter studies^34^. Based on our gene list, NanoString Technologies, Inc. (Seattle, WA) designed the optimized 50-base nCounter gene-specific capture probes for the platform using proprietary methods based upon transcript frequency. A full list of gene symbols and the probe design for these 536 genes can be found in Supplementary Table S2. This multiplexed assay is reported to detect expression of up to 800 transcripts at very low mRNA concentrations (0.1 f M/1 copy per cell)^32^ even in RNA samples that are significantly degraded, as long as at least 20% of the sample has RNA fragments of 300 base pairs or longer^35^. Therefore, the 194 CRC samples with 20% to 91% (median value is 37%) of the RNA sample containing fragments of 300 base pairs or longer in length were hybridized to the custom nCounter^®^ assay. To examine the reproducibility of the nCounter platform, we generated one more set of technical replicates (RNA samples from the same extraction) for 32 samples and measured their gene expression using nCounter platform for inter-assay comparisons.

### Gene expression profiling based on HTA platform

After performing gene expression profiling of all 194 FFPE tumor samples on the nCounter platform, 89 of the 194 samples had sufficient sample remaining for subsequent gene expression profiling based on the Affymetrix HTA platform. Among these 89 samples, 61 samples are technical replicates (mRNA samples from the same extraction) and 28 are biological replicates (mRNA samples from the same FFPE tumor block but different extractions). For gene expression analysis using FFPE-derived RNA samples, Illumina (llumina Inc., San Diego, CA) recommends using RNA samples containing at least 30% 200-base pair or longer RNA fragments^36^. Using this criterion, we removed samples with less than 30% 200 base-pair RNA fragments or labeled as tube almost empty and this process yielded 84 samples with 43% to 95% (median value is 65.5%) 200-base pair or longer RNA fragments. Finally, we performed gene expression profiling for the 84 of the 194 FFPE-derived CRC tissues using GeneChip^®^ Human Transcriptome Array 2.0 (Affymetrix, Santa Clara, CA). These 84 samples had also been evaluated by the nCounter assay as described above.

### Data preprocessing

For the NanoString nCounter data, samples with average count ≤1000 and missing rate (the percentage of genes with the average expression count ≤ the average count of a synthetic negative control gene set +3 standard deviations of negative control gene set) ≥20% based on raw count data were removed to ensure data quality. Then, using R package *NanoStringNorm*^37^, we performed quality control (remove samples with low counts in positive control genes, high counts in negative control genes and high counts in House Keeping genes) and normalization. The batch effects were adjusted by the ComBat approach available in the Bioconductor package *SVA*^38^. Negative values caused by batch effect adjustment were replaced by 0.

For HTA data, we normalized the raw data at the gene level using Robust MultiChip Averaging (RMA) algorithm as implemented in the Affymetrix^®^ Expression Console^TM^ Software 1.3 and then mapped the probe set identifiers to gene symbols based on the annotation file downloaded within the Expression Console application. Probe sets mapped to multiple genes were eliminated. When multiple probe sets were mapped to the same gene symbol, the probe set with largest interquartile range was kept. Then, we adjusted the batch effects using the ComBat function available in the Bioconductor package *SVA*^38^. Negative values caused by batch effect adjustment were replaced by 0.

In this study, in order to make direct comparison between nCounter and HTA gene expression data, we summarized both datasets at the gene level and used gene symbol to link NanoString nCounter IDs and Affymetrix Probe IDs.

### Quality of gene expression data measured by nCounter and HTA

**Figure 1:**
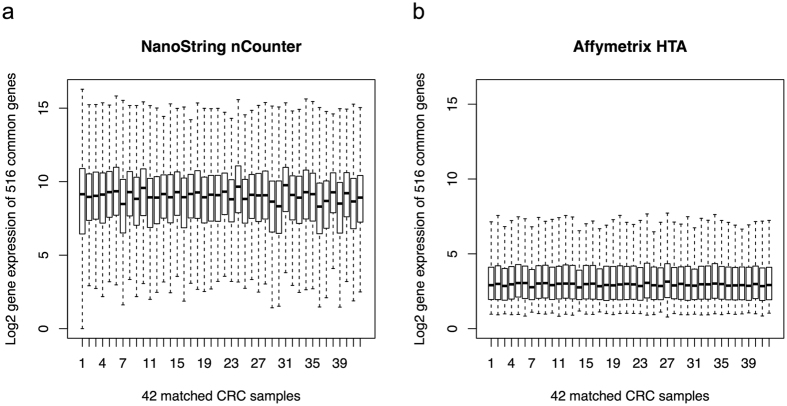
The signal intensity distributions of the 42 pairs of matched CRC samples measured by both nCounter platform and HTA platform. The distributions of signal intensities of 42 pairs of matched CRC samples (30 technical replicates and 12 biological replicates) measured by nCounter platform (**a**) and HTA platform (**b**).

After data pre-processing, 99 out of 194 CRC samples measured by nCounter platform and all the 84 CRC samples measured by HTA platform have gene expression data with sufficient quality for comparative analysis. Among these remaining samples, 42 CRC samples and 516 genes were measured by both platforms. These 42 pairs of CRC samples contained 30 pairs of technical replicates (mRNA samples from the same extraction, the FFPE block storage time from date of resection to RNA extraction is the same for samples measured by both platforms) and 12 pairs of biological replicates (mRNA samples from the same FFPE tumor block but different extractions, the FFPE block storage time from date of resection to RNA extraction is one year later for samples measured by HTA platform than that for samples measured by nCounter platform) (Supplementary Table S3). The distributions of signal intensities (normalized and log2 transformed gene expression data) for 42 pairs of matched samples across 516 common genes are displayed with boxplots in Fig. 1. The expression patterns of the 42 common samples within each platform were comparable and had different scales between different platforms. It should be noted that the difference in the absolute gene expression values from these two platforms is due to the different measurement scales on these two platforms, not indicating different data quality. No obvious outlier with extremely small interquartile range was observed.

To examine the reproducibility of the nCounter platform, we generated one more set of technical replicates (mRNA samples from the same extraction) for 32 samples and measured their gene expression using the nCounter platform. To control for the quality of the nCounter data, samples with average count greater than 1000 and less than 20% of genes with the average expression count ≤ average count of a synthetic negative control gene set +3 standard deviations of negative control gene set were selected. Among these 32 pairs of technical replicates, five pairs of technical replicates have gene expression data with sufficient quality for correlation analysis. The scatterplots of the normalized counts between the each pair of the replicates with the Pearson correlation coefficient (r) were shown in Supplementary Fig. S1. The high correlation (r = 0.95–0.97) indicates that the gene expression data measured by nCounter platform are reproducible after data quality control. However, only 15.6% of the replicate pairs passed the quality control parameters.

### Sample-wise and gene-wise correlations between gene expression data measured by nCounter platform and HTA platform

**Figure 2:**
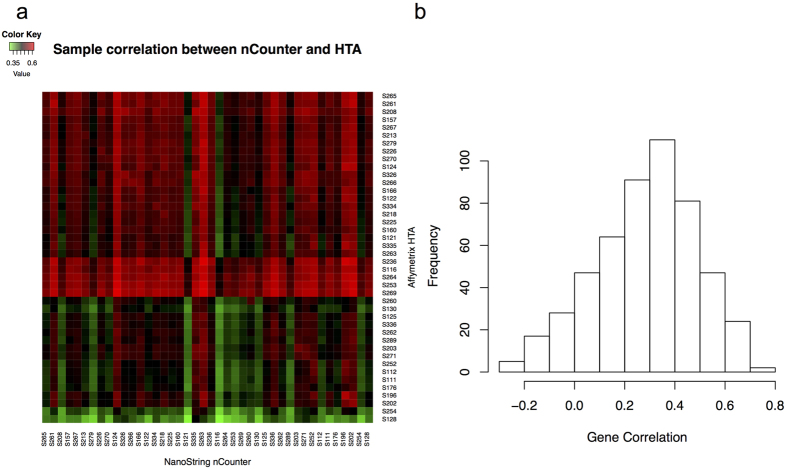
The sample-wise and gene-wise correlations based on 42 pairs of matched CRC samples. (**a**) The heatmap shows all the pairwise correlations (Pearson correlation coefficients) between the 42 pairs of matched samples measured by nCounter and HTA platforms. (**b**) The histogram shows the gene-wise correlations (Pearson correlation coefficients) for the 516 common genes measured by both nCounter and HTA platforms across 42 pairs of matched CRC samples.

For each of the 42 pairs of FFPE-derived CRC samples measured by both nCounter and HTA, we calculated the Pearson correlation coefficient based on normalized, log2 transformed gene expression values across 516 genes that were common to both platforms. The Affymetrix GeneChip^®^ Human Transcriptome Array 2.0 covers the entire transcriptome (25095 genes), however, we restricted the analytical comparison to only those genes that were included in the nCounter codeset. The mean correlation between 42 pairs of matched CRC samples is 0.51 with 95% bootstrap confidence interval (0.49, 0.53), while the maximum and minimum correlations are 0.59 and 0.38, respectively. The heatmap in Fig. 2a displays all the pairwise correlations between the 42 pairs of matched CRC samples measured by both nCounter and HTA platforms.

In addition to sample-wise correlation, we also calculated the Pearson correlation coefficient for each of the 516 genes across 42 common samples measured by both nCounter and HTA platforms. As shown in Fig. 2b, the mean gene-wise correlation coefficient of the 516 genes is 0.29 with 95% bootstrap confidence interval (0.27, 0.3), while the maximum and minimum correlations are 0.79 and −0.28.

### Evaluation of the frozen tissue-derived prognostic signatures based on the nCounter platform and the HTA platform

**Figure 3:**
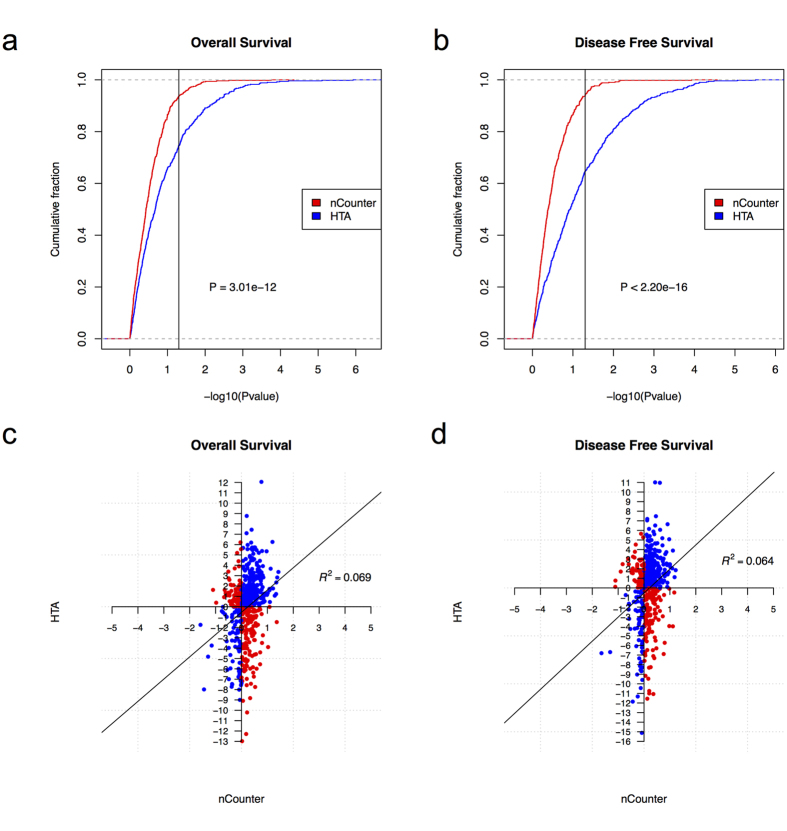
Association between gene expression patterns and clinical outcomes based on nCounter platform and HTA platform. The association between gene expression and five-year overall survival (OS) and disease free survival (DFS) were measured by Cox proportional hazard model and represented by the resulted P values and log Hazard ratios. The cumulative distribution functions for −log10(Pvalue) of 516 common genes from both nCounter platform (red) and HTA platform (blue) were shown in (**a**) for OS and (**b**) for DFS. The vertical lines in (**a,b**) represent P value 0.05 on the x-axis. P values for two-sample Kolmogorov-Smirnov test were shown in (**a,b**). Scatterplots of log Hazard ratios for 516 common genes from both nCounter platform and HTA platform were shown in (**c**) for OS and (**d**) for DFS. R^2^ from linear fitting were shown in (**c,d**). Blue dots represent genes with positive (or negative) log Hazard ratios based on both platforms. Red dots represent genes with positive log Hazard ratios based on one platform but negative log Hazard ratios based on the other platform.

To evaluate the frozen tissue-derived prognostic signatures in FFPE-derived CRC tissues, we examined the association of gene expression with 5-year overall survival (OS) outcomes and 5-year disease free survival (DFS) outcomes for each of the 516 common genes using a Cox proportional hazard model. After extracting the P values of the log-rank tests from the Cox proportional hazard models, we compared the cumulative distribution functions (CDF) for the -log10 (P value) of the 516 common genes from both platforms. As shown in Fig. 3a, for the association with OS, the CDF of the −log10 (P value) for the nCounter data lies above that of the HTA data (P = 3.01e–12, one-sided Kolmogorov-Smirnov test) and the increase of the CDF of the −log10 (Pvalue) of the HTA data is higher than that of the nCounter data after P = 0.05 (the vertical line). This result indicates that more signature genes associated with OS can be found using HTA data with P value < 0.05 based on these 516 common genes. Evaluation of the gene expression data and the association with DFS as shown in Fig. 3b, again demonstrates that the CDF of the −log10 (P value) for the nCounter data lies above that of the HTA data (P < 2.2e–16, one-sided Kolmogorov-Smirnov test) and the increase of the CDF of the −log10 (P value) of the HTA data is much larger than that of the nCounter data after P = 0.05 (the vertical line). Similar to the OS results, more signature genes associated with DFS can be found using HTA data with P value < 0.05 based on these 516 common genes (see Cox regression results in Supplementary Table S4).

In addition to p values, we also compared the log hazard ratios for the 516 common genes based on nCounter and HTA data. As shown in Fig. 3c,d, for the association with both OS and DFS, there is no correlation between the log hazard ratios computed based on nCounter data and HTA data for the same gene. Moreover, among the 516 genes compared across both platforms, 194 genes show opposite direction of association with OS outcomes based on data from the two platforms (red dots in Fig. 3c), while 188 genes show opposite direction of association with DFS outcomes based on data from these two platforms (red dots in Fig. 3d).

**Figure 4:**
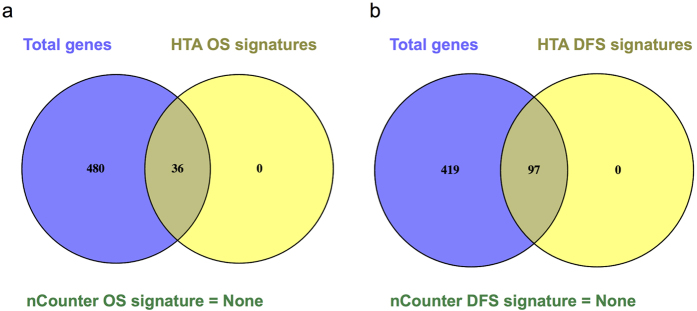
Overlapping of genes significantly associated with clinical outcomes based on nCounter platform and HTA platform. The association between gene expression and five-year OS and DFS were measured by Cox proportional hazard model. (**a**) Using a false discovery rate 0.05 as the threshold, there are 36 of 516 genes significantly associated with OS based on HTA platform while no gene was found to be significantly associated with OS based on nCounter platform. (**b**) With the same threshold, there are 97 of 516 genes significantly associated with DFS based on HTA platform, while no gene was found to be significantly associated with DFS based on nCounter platform.

We next examined the subsets of genes (of the 516 total) significantly associated with clinical outcomes based on the nCounter platform and the HTA platform. Using a false discovery rate (FDR) 0.05 as the threshold, 36 of the 516 common genes are significantly associated with OS based on the HTA platform (FDR < 0.05, log-rank test) while no individual gene of the 516 common genes was found to be significantly associated with OS based on the nCounter platform (Fig. 4a). With the same threshold, 97 of the 516 common genes are significantly associated with DFS based on the HTA platform. Similarly, no individual gene is significantly associated with DFS based on the nCounter analysis data (Fig. 4b). By combing the 36 genes significantly associated with OS and 97 genes significantly associated with DFS, we identified 99 unique genes whose up- or down- regulations are significantly associated with poor survival (DFS and/or OS). Among these 99 genes, 48 genes are from multi-gene prognostic signatures with up- or down- regulation information available from the original papers. We found that 29 of the 48 genes showed gene expression changes associated with survival in the same direction across studies using frozen tissue samples and FFPE samples (hazard ratio (HR) >1 (<1) based on data from FFPE samples and up (down) regulated in the patients with tumor recurrence in the previous frozen sample based study).

In addition to examining the single-gene level signatures, we also evaluated the performance of previously reported frozen tissue-derived multi-gene prognostic signatures in FFPE derived samples based on the nCounter platform and the HTA platform. In total, six previously reported multi-gene signatures have sufficient information from the original papers enabled such evaluation. First, we removed genes showing gene expression changes associated with survival in the opposite directions across studies using frozen tissue samples and FFPE samples in each multi-gene signature. During this process, different genes might be filtered based on HTA platform-derived data and nCounter platform-derived data, separately. Then, on the basis of the average of median normalized gene expression levels of each multi-gene signature, we classified patients into two groups (high risk group with above median expression and low risk group with below median expression).

**Figure 5:**
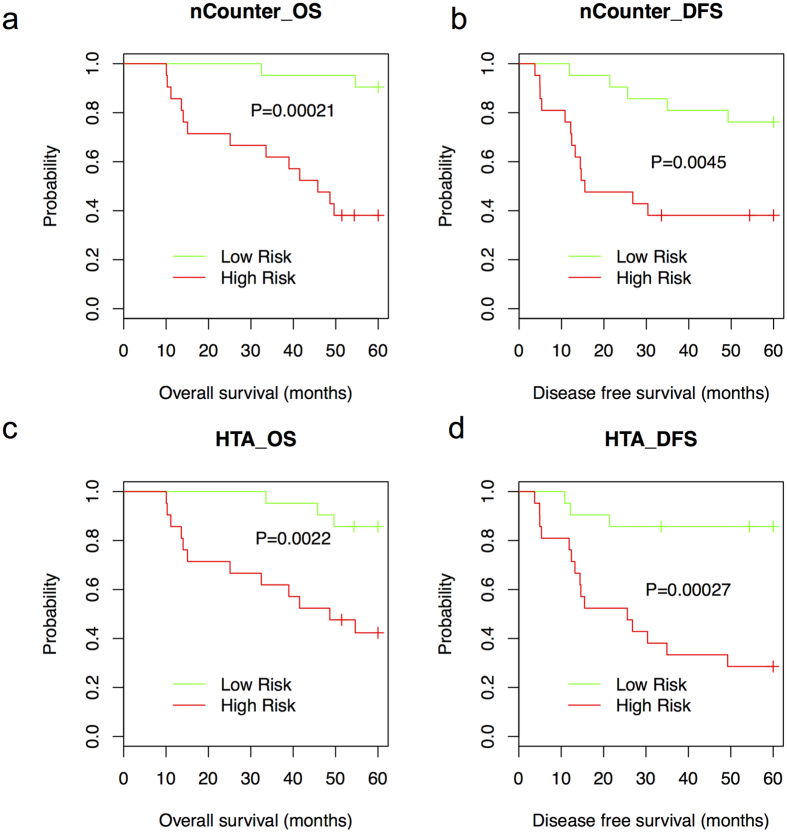
Performance of frozen tissue-derived prognostic signature from Wang, Y. *et al.* in FFPE-derived samples based on nCounter platform and HTA platform. Kaplan-Meier estimates of OS (**a,c**) and DFS (**b,d**) according to the risk prediction by gene signature from Wang, Y. *et al.* based on nCounter platform (7 of 10 available genes) and HTA platform (5 of 10 available genes, 42 samples were divided into two groups with 21 samples in each group).

**Figure 6:**
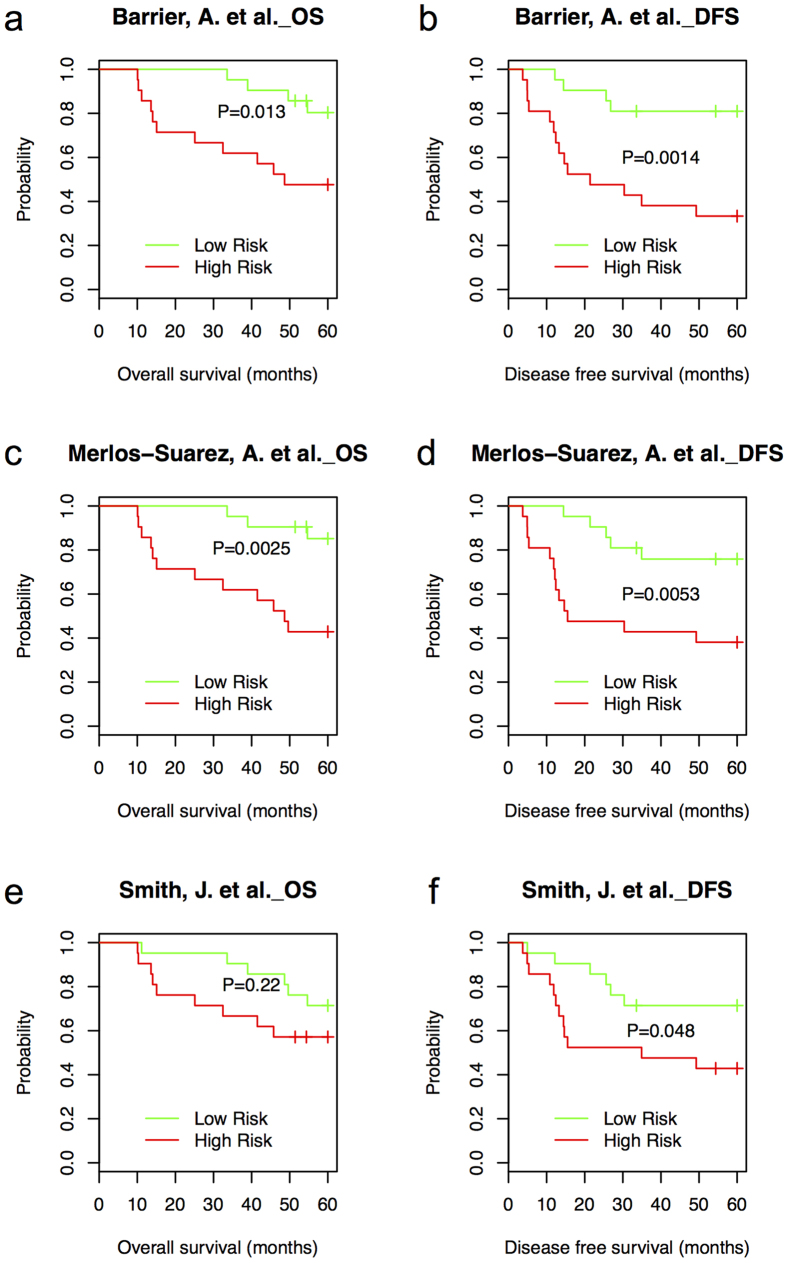
Performance of frozen-tissue derived prognostic signatures from Barrier, A. *et al.,* Merlos-Suarez, A. *et al.* and Smith, J. J. *et al.* in FFPE-derived samples based on HTA platform. Kaplan-Meier estimates of OS (**a**,**c** and **e**) and DFS (**b**,**d** and **f**) according to the risk prediction by gene signatures from Barrier, A. *et al.* (22 of 34 available genes), Merlos-Suarez, A. *et al.* (6 of 9 available genes) and Smith, J. *et al.* (23 of 31 available genes) based on HTA platform (42 samples were divided into two groups with 21 samples in each group).

Kaplan-Meier estimates of OS and DFS showed that based on HTA platform-derived data, four of the six available multi-gene signatures from Wang, Y. *et al.*^30^, Barrier, A. *et al.*^17^, Merlos-Suarez, A. *et al.*^25^ and Smith, J. J. *et al.*^28^ can divide patients into high-risk and low-risk groups with significantly different DFS (P < 0.05, log-rank test, Figs 5d and 6b,d,f) and three of the four multi-gene signatures from Wang, Y. *et al.*^30^, Barrier, A. *et al.*^17^ and Merlos-Suarez, A. *et al.*^25^ can divide patients into high-risk and low-risk groups with significantly different OS (P < 0.05, log-rank test, Figs 5c and 6a,c). Based on nCounter platform-derived data, one of the six published multi-gene signatures from Wang, Y. *et al.*^30^ can divide patients into high-risk and low-risk groups with significantly different DFS and OS (P < 0.05, log-rank test, Fig. 5a,b). The performances of multi-gene signatures from Barrier, A. *et al.*^17^, Merlos-Suarez, A. *et al.*^25^ and Smith, J. J. *et al.*^28^ based on nCounter platform-derived data are shown in Figure S2. When checking the overlapping patients from high/low-risk groups predicted by gene signatures from Wang, Y. *et al.*^30^, Barrier, A. *et al.*^17^, Merlos-Suarez, A. *et al.*^25^ and Smith, J. J. *et al.*^28^ based on HTA and nCounter platform, separately, we found 71%, 52%, 57% and 52% of the total patients were classified into the same risk group (Supplementary Tables S6–9).

The four multi-gene signatures from Wang, Y. *et al.*^30^, Barrier, A. *et al.*^17^, Merlos-Suarez, A. *et al.*^25^ and Smith, J. J. *et al.*^28^, which are filtered based on HTA platform-derived data and nCounter platform-derived data, separately and used in this study are shown in Supplementary Table S5. In addition, we also evaluated the association between gene expression patterns and clinical outcomes for multi-gene signatures with sufficient information from the original papers without filtering. As shown in Supplementary Table S10, two multi-gene signatures from Merlos-Suarez, A. *et al.*^25^ and Smith, J. J. *et al.*^28^ can divide patients into high-risk and low-risk groups with significantly different DFS (P ≤ 0.05, log-rank test) based on HTA platform and one multi-gene signature from Barrier, A. *et al.*^17^ can divide patients into high-risk and low-risk groups with significantly different OS (P ≤ 0.05, log-rank test) based on nCounter platform. This result highlights the robustness of the multi-gene signatures from Merlos-Suarez, A. *et al.*^25^, Smith, J. J. *et al.*^28^ and Barrier, A. *et al.*^17^ in dividing patients into groups with significantly different DFS or OS.

## Discussion

Performing gene expression profiling for FFPE samples is crucial for developing robust gene expression signatures that could be readily translated through testing in clinical trials. Nanostring nCounter^®^ and Affymetrix GeneChip^®^ Human Transcriptome Array 2.0 are the two available platforms that enable targeted and genome wide gene expression profiling of FFPE samples, separately. In this study, based on these two platforms, we evaluated the prognostic value of 516 genes that were derived from multiple published frozen tissue-derived CRC prognostic signatures and present on both platforms.

Based on HTA platform-derived data, our results showed that (1) 36 and 97 of the 516 common genes are significantly associated with OS and DFS at the single gene level (FDR < 0.05, log-rank test), respectively; (2) 60% of these survival associated genes which have direction information from the original papers showed gene expression changes associated with survival in the same direction across studies using frozen tissue samples and FFPE samples; and (3) four of the six reported multi-gene signatures, for which sufficient information from the original papers enabled such evaluation, can divide patients into high-risk and low-risk groups with significantly different DFS (P < 0.05, log-rank test) and three of the four multi-gene signatures can divide patients into high-risk and low-risk groups with significantly different OS (P < 0.05, log-rank test). Based on nCounter platform-derived data, no individual gene was found to be significantly associated with survival at the single gene level (FDR < 0.05, log-rank test), but one of the six published multi-gene signatures can divide patients into two groups with significantly different DFS and OS (P < 0.05, log-rank test). These results indicate that sufficiently high quality RNA could be obtained from FFPE tumor tissues to detect frozen tissue-derived prognostic gene expression signatures for CRC patients. According to Table S1, most of the public signatures were identified based on gene expression microarray (mainly used Affymetrix platforms). To measure gene expression, microarray requires cDNA synthesis, labeling, hybridization, and intensity measurement. Then, to reduce the technical variations and biases introduced in each step, normalization is required before comparisons between samples^39^. A popular method, RMA including background correction, quantile normalization, and expression summarization for each probe set using median polish on a linear model^40^ is frequently used for Affymetrix arrays normalization^39^. In contrast, the nCounter System directly measures the mRNA expression levels by dual probe hybridization without RNA amplification. The difference between microarray and the nCounter technology might cause the better performance in rediscovering the previously published prognostic signatures identified mainly using microarray.

In our previous study, we found moderate sample-wise correlation between paired FFPE samples measured by the nCounter platform and fresh-frozen samples measured by the Affymetrix Human Genome U133 Plus 2.0 platform^34^. Similarly, in the current study, we also observed moderate correlations between paired FFPE samples measured by the nCounter and HTA platforms (mean Pearson correlation coefficient is 0.51, mean Spearman correlation coefficient is 0.54). Since nCounter used single probe designed for each transcript while HTA used multiple-probe design, the different probe design methods might cause the moderate correlations between sample pairs (also between gene pairs) in the cross platform comparisons. Although different normalization methods were used for different platforms, this should not change the gene ranking within each sample. Thus, the correlation pattern between samples will not be affected by different normalization methods. A previous study showed a high correlation coefficient (r = 0.9) between matched fresh-frozen and FFPE samples when both samples were measured by the nCounter platform^35^. In this study, we also found high correlation between the gene expressions of FFPE-derived technical replicates both measured by the nCounter platform.

The degree of RNA degradation is a challenging issue in gene expression analysis using FFPE-derived samples. As shown in the Supplementary Table S3, the median storage times of the paraffin blocks of tissues (from the date of resection to the date of RNA extraction) for the 42 pairs of FFPE-derived samples measured by the nCounter platform and the HTA platform are 14.5 years and 15.5 years. The technical replicates were extracted at the same time (same storage time) and the biological replicates were obtained one year later. To reduce the effect of RNA degradation, we used a consistent procedure for sample preparation and conducted strict quality control analysis before submitting samples (Supplementary Table S3). In addition, the inherent tumor heterogeneity might also cause the moderate sample wise correlation. In our study, the median sample wise correlation between 30 pairs of technical replicates is 0.53, while the median sample wise correlation between 12 pairs of biological replicates is 0.51 (the difference is not statistically significant).

Since the surgical specimens are routinely preserved by fixation in formalin, archived FFPE tumor tissues remain the most available tissue specimen source for biomarker study in CRC, at least on a retrospective basis, and for practical purposes on any large-scale multi-center prospective study. Our study showed that sufficiently high quality RNA can be obtained from FFPE preserved archival tumor tissues to detect gene expression signatures originally described from fresh frozen tissues with consistent expression patterns in FFPE tissues and correlation with clinical outcomes for CRC patients. For example, frozen tissue derived gene expression signatures from Wang, Y. *et al.*^30^, Merlos-Suarez, A. *et al.*^25^ and Barrier, A. *et al.*^17^ could divide patients into high-risk and low-risk groups with significantly different DFS and OS based on their gene expression patterns in FFPE tissues (P < 0.05, log-rank test). Our previously identified 34-gene prognostic signature using frozen tissues^28^ could divide patients into high-risk and low-risk groups with significantly different DFS based on gene expression patterns in FFPE tissues (P < 0.05, log-rank test). The difference of OS between the high-risk and low-risk groups is not statistically significant (P = 0.22) that may be due to the small sample size (n = 21 in each group). We will further test the association with OS by increasing the sample size in a future study.

Our study provides interesting prognostic gene expression signature candidates that show consistent association with clinical outcomes across frozen and FFPE CRC tissues. This work needs to be further validated in larger cohorts of FFPE samples. Improvements in technology such as the single cell level RNA sequencing approaches^41^ might increase the percentage of genes robustly detected as biomarkers across both frozen and FFPE tumor tissues. This work provides a path for validation of gene expression signatures in large numbers of FFPE tumor tissue samples that are annotated by patient treatments and outcomes in order to identify both prognostic and predictive gene expression biomarkers to help guide treatment decisions.

## Methods

### Sample preparation

Human CRC tissues were collected and annotated according to established protocols and approved by the appropriate Institutional Review Boards (IRB) at Vanderbilt University Medical Center (VUMC). All tissues were collected over the time period from 1999–2011. Tumor stage was assessed by American Joint Commission on Cancer (AJCC) 7^th^ Edition guidelines^42^. Written informed consent has been obtained from the subjects since 2003. A waiver of consent under Vanderbilt University IRB#101531 allowed inclusion of cases from 1983 to 2003 who did not consent. Study inclusion was determined by appropriate IRB approved investors and this information was de-identified and HIPAA-compliant prior to release of data to key study personnel.

Archived CRC FFPE samples used in this study were stored at room temperature until sectioned for RNA extraction. The first 10 μm of the tissue was discarded before cutting sections for RNA extraction. Five-micron sections were mounted on uncharged glass slides. Separate tissue sections from the top and bottom of the serial sections used for RNA extraction were stained with H&E for quality control from each tissue block. RNA was purified from 5 μm thick tissue sections containing greater than 80% tumor using High Pure FFPE RNA Micro Kit (Roche) according to manufacturer’s instructions. A minimum of 4 sections per sample were required. After extraction, total RNA samples were submitted to Vanderbilt Technologies for Advanced Genomics for quality control analysis by an Agilent Bioanalyzer instrument.

### Data Analysis

We examined the association of gene expression with five-year OS and DFS outcomes using the Cox proportional hazard model available in the R package *survival*. The P values are from log-rank tests. The resulting P values were adjusted for multiple-hypothesis testing using the Benjamini-Hochberg method^43^. All the analyses were performed in R (version 3.1.0). Both nCounter and HTA data are available in Gene Expression omnibus (GEO) database (GSE78248).

## Additional Information

**How to cite this article**: Zhu, J. *et al.* Evaluation of frozen tissue-derived prognostic gene expression signatures in FFPE colorectal cancer samples. *Sci. Rep.* **6**, 33273; doi: 10.1038/srep33273 (2016).

## Supplementary Material

Supplementary Information

Supplementary Table S1-S10
