# Supplementary material for: Evaluation of frozen tissue-derived prognostic gene expression signatures in FFPE colorectal cancer samples
Source: Sci Rep. 2016 Sep 14;6:33273. doi: 10.1038/srep33273 (PMC5021945; doi:10.1038/srep33273)
Supplement: Supplementary Information [file srep33273-s1.pdf]

Evaluation of frozen tissue-derived prognostic gene expression signatures in FFPE colorectal cancer samples

Jing Zhu[1,2], Natasha G. Deane[1,2], Keeli B. Lewis[1], Chandrasekhar Padmanabhan[1], M. Kay Washington[2,3], Kristen K. Ciombor[4], Cynthia Timmers[4], Richard M. Goldberg[4], R. Daniel, Beauchamp[1,2,5,6,*], Xi Chen[7,8,*]

1. Vanderbilt University, Department of Surgery, Nashville, 37232, USA
2. Vanderbilt University, Vanderbilt Ingram Cancer Center, Nashville, 37232, USA
3. Vanderbilt University, Department of Pathology, Nashville, 37232, USA
4. The Ohio State University Comprehensive Cancer Center, Columbus, 43210, USA
5. Vanderbilt University, Department of Cell and Developmental Biology, Nashville, 37232, USA
6. Vanderbilt University, Department of Cancer Biology, Nashville, 37232, USA
7. University of Miami Miller School of Medicine, Division of Biostatistics, Department of Public Health Sciences, Miami, 33136, USA
8. University of Miami Miller School of Medicine, Sylvester Comprehensive Cancer Center, Miami, 33136, USA

[*] steven.chen@miami.edu and daniel.beauchamp@vanderbilt.edu

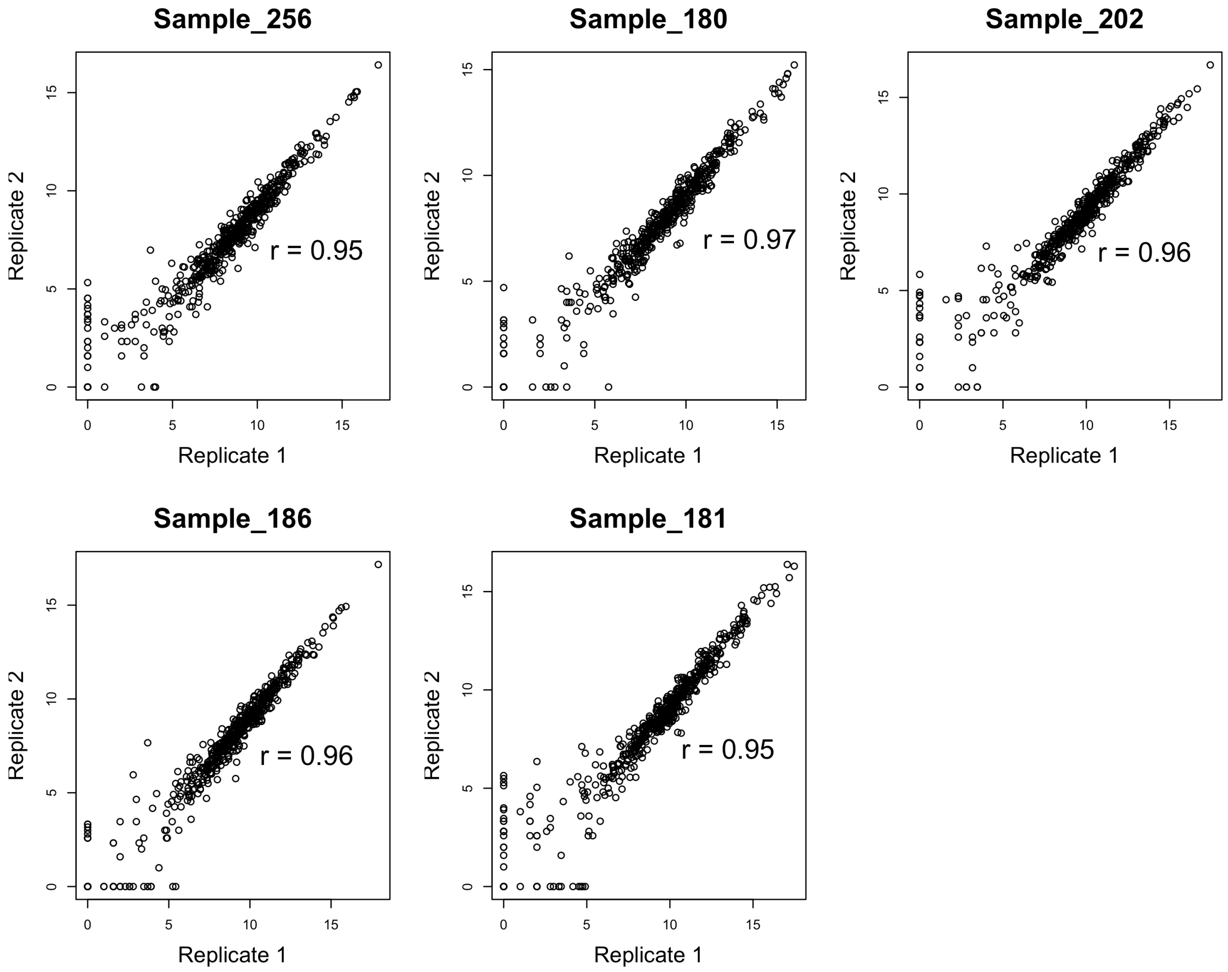

Supplementary Figure S1. Scatterplot of the normalized counts from 536 individual gene elements using a split sample from single FFPE preserved tumor extraction on the nCounter platform. r shows the Pearson correlation coefficient.

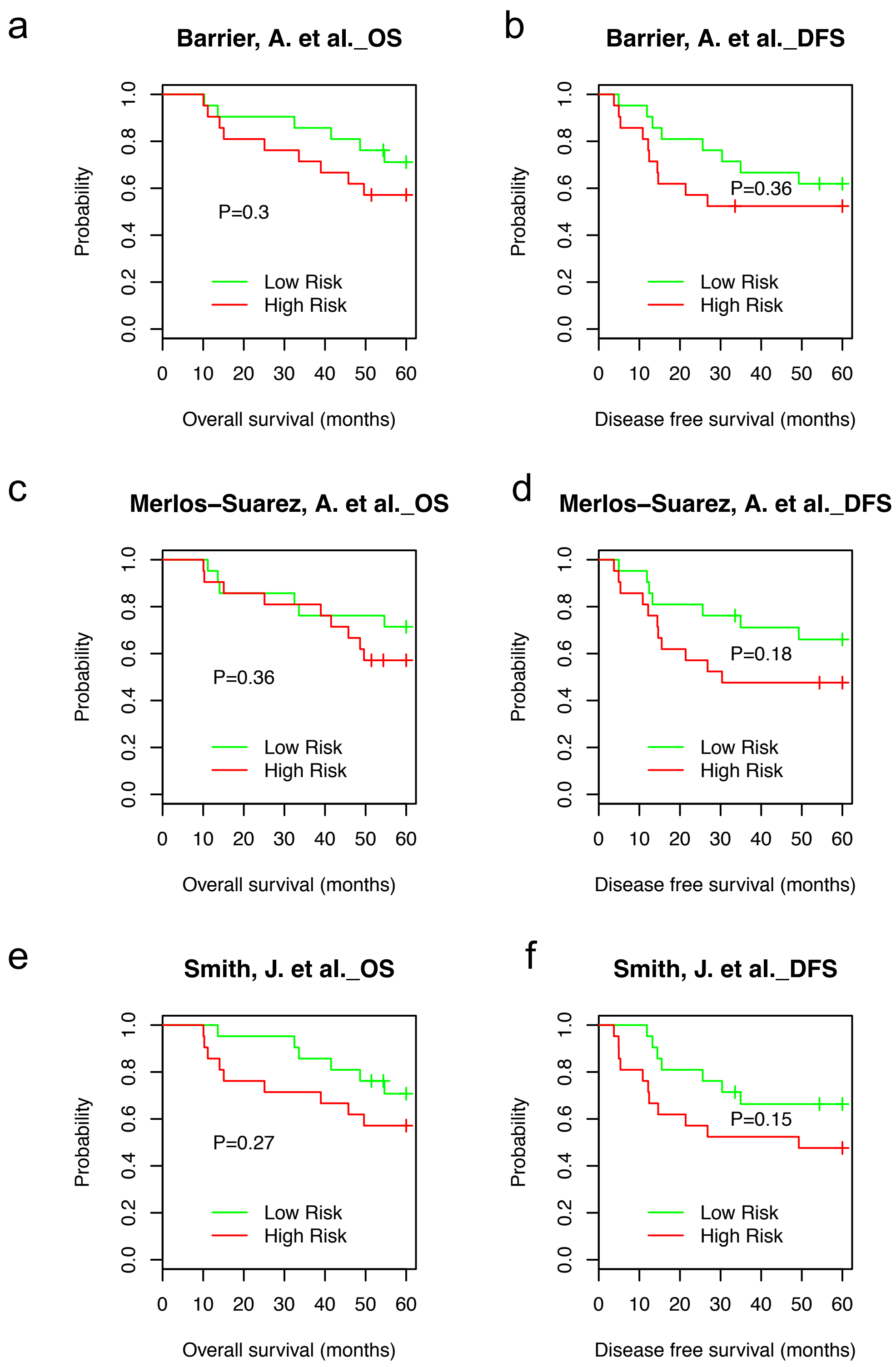

Supplementary Figure S2. Performance of frozen-tissue derived prognostic signatures from Barrier, A. *et al.*, Merlos-Suarez, A. *et al.* and Smith, J.J. *et al.* in FFPE-derived samples based on nCounter platform. Kaplan-Meier estimates of OS (a, c and e) and DFS (b, d and f) according to the risk prediction by gene signatures from Barrier, A. *et al.* (19 of 34 available genes), Merlos-Suarez, A. *et al.* (5 of 9 available genes) and Smith, J.J. *et al.* (13 of 31 available

genes) based on nCounter platform (42 samples were divided into two groups with 21 samples in each group).
